# Selective Inhibition of Prostasin in Human Enterocytes by the Integral Membrane Kunitz-Type Serine Protease Inhibitor HAI-2

**DOI:** 10.1371/journal.pone.0170944

**Published:** 2017-01-26

**Authors:** Frank Shiao, Li-Ching O. Liu, Nanxi Huang, Ying-Jung J. Lai, Robert J. Barndt, Chun-Che Tseng, Jehng-Kang Wang, Bailing Jia, Michael D. Johnson, Chen-Yong Lin

**Affiliations:** 1 Lombardi Comprehensive Cancer Center, Department of Oncology Georgetown University Washington DC, United States of America; 2 College of Medicine, National Defense Medical Center, Taipei, Taiwan; 3 Department of Biochemistry, National Defense Medical Center, Taipei, Taiwan; 4 Department of Gastroenterology, Henan Provincial People’s Hospital, Zhengzhou, China; Florida State University, UNITED STATES

## Abstract

Mutations of hepatocyte growth factor activator inhibitor (HAI)-2 in humans cause sodium loss in the gastrointestinal (GI) tract in patients with syndromic congenital sodium diarrhea (SCSD). Aberrant regulation of HAI-2 target protease(s) was proposed as the cause of the disease. Here functional linkage of HAI-2 with two membrane-associated serine proteases, matriptase and prostasin was analyzed in Caco-2 cells and the human GI tract. Immunodepletion-immunoblot analysis showed that significant proportion of HAI-2 is in complex with activated prostasin but not matriptase. Unexpectedly, prostasin is expressed predominantly in activated forms and was also detected in complex with HAI-1, a Kunitz inhibitor highly related to HAI-2. Immunohistochemistry showed a similar tissue distribution of prostasin and HAI-2 immunoreactivity with the most intense labeling near the brush borders of villus epithelial cells. In contrast, matriptase was detected primarily at the lateral plasma membrane, where HAI-1 was also detected. The tissue distribution profiles of immunoreactivity against these proteins, when paired with the species detected suggests that prostasin is under tight control by both HAI-1 and HAI-2 and matriptase by HAI-1 in human enterocytes. Furthermore, HAI-1 is a general inhibitor of prostasin in a variety of epithelial cells. In contrast, HAI-2 was not found to be a significant inhibitor for prostasin in mammary epithelial cells or keratinocytes. The high levels of constitutive prostasin zymogen activation and the selective prostasin inhibition by HAI-2 in enterocytes suggest that dysregulated prostasin proteolysis may be particularly important in the GI tract when HAI-2 function is lost and/or dysregulated.

## Introduction

Autosomal-recessive congenital sodium diarrhea (CSD) is a rare, inherited diarrhea of infancy, characterized by watery diarrhea with extraordinarily high fecal sodium loss [1,2]. Studies of the clinical phenotype and the molecular basis of the condition using the positional candidate approach has identified a subtype of CSD, known as the syndromic form of CSD (SCSD), which differs from classic CSD by its association with choanal or anal atresia, hypertelorism, and corneal erosions not found with classic CSD [3,4]. Five distinct mutations of *SPINT2* have been identified in SCSD but none in classic CSD [3]. *SPINT2* encodes an integral membrane, Kunitz-type serine protease inhibitor, known as hepatocyte growth factor activator inhibitor (HAI)-2 or placenta bikunin [5,6]. The mutations associated with SCSD appear to result in either loss of protein synthesis or reduced anti-protease activity of HAI-2, both of which theoretically result in the increased proteolytic activity of HAI-2 target proteases in the affected tissues. Alternatively, the loss of HAI-2 function may result in suppression of the synthesis, intracellular trafficking, and even zymogen activation of target proteases, resulting in a paradoxical reduction in the proteolytic activity of HAI-2 target proteases. This scenario has been observed with the type 2 transmembrane serine protease matriptase, whose expression, intracellular trafficking, and zymogen activation depends on the inhibitor HAI-1 in some systems [7,8]. Whether one of these or some other scenario is involved, the underlying mechanism by which HAI-2 mutation causes sodium loss in SCSD remains elusive because it remains unclear which serine protease(s) are impacted by HAI-2 mutation in this context.

Several serine proteases have been identified as the potential target proteases of HAI-2, including hepatocyte growth factor activator (HGFA), hepsin, trypsin, plasma kallikrein, plasmin, factor XIa, matriptase and prostasin [5,6,9,10]. The characterization of these enzymes as HAI-2 target proteases has, however, largely been conducted in solution with purified HAI-2 and/or these serine proteases, and so whether in fact they are all true physiological targets of HAI-2 remains to be determined. More recently, HAI-2 has been purified from human milk in complexes with the activated forms of matriptase or the GPI-anchored serine protease prostasin [11]. The presence of these protease-protease inhibitor complexes in normal body fluid implies that matriptase and prostasin are physiological targets of HAI-2, at least during lactation. The lack of free HAI-2 or free active matriptase in human milk, suggests that the formation of the HAI-2-matriptase or -prostasin complexes occurs in lactating mammary epithelial cells prior to their shedding into the milk. If active matriptase were shed to the milk separate from HAI-2 and the complexes formed subsequently in solution, it seems likely that some levels of the monomeric forms would be detectable. In addition to HAI-2, both matriptase and prostasin are also present in complexes with HAI-1 in human milk. An *in vivo* functional link between these two membrane-bound serine proteases and the two Kunitz inhibitors has also been observed in several animal studies. In the mouse and/or zebra fish, epidermal, placental or embryonic defects associated with genetic ablation of HAI-1 or HAI-2 can be rescued by simultaneous deletion or suppression of matriptase or prostasin [12–16].

HAI-2, HAI-1, matriptase, and prostasin are widely expressed in a variety of epithelial tissues [5,10,17–19]. In contrast to matriptase which is localized primarily at the cell surface, HAI-2 has been consistently detected in punctate intracellular granules/vesicles-like structures in cultured human mammary epithelial cells [20,21] and in tissue sections of human intestine epithelium [22]. The inhibition of matriptase by HAI-2 can occur when a proportion of the HAI-2 is translocated to the cell surface, as has been observed in some breast cancer cells [20]. It seems that inhibitory activity of HAI-2 against given target proteases depends on whether they are in a similar subcellular localization and this may occur in a cell type-selective fashion. This is in a stark contrast to the situation with HAI-1, where its activity as a matriptase inhibitor is ubiquitous in matriptase-expressing epithelial and carcinoma cells, most likely due to their similar subcellular localization. In the current study, we set out to identify and characterize the HAI-2 target protease in Caco-2 cells and human intestinal tissues with the goal of gaining insights into the molecular mechanism underlying the defect in sodium homeostasis in the GI tract caused by the loss of HAI-2 function.

## Materials and Methods

### Chemicals and reagents

5,5’-Dithio-bis-(2-Nitrobenzoic Acid) (DTNB) was obtained from Sigma-Aldrich (St. Louis, MO); the prostasin fluorogenic substrate Ac-Lys-Tyr-His-Arg-7-Amido-4- methylcoumarin (Ac-KYHR-AMC) [23] was purchased from AnaSpec Inc. (Fremont, CA).

#### Cell cultures

Caco-2 (ATCC, Manassas, VA) is a cell line derived from a human colorectal adenocarcinoma. These cells are widely used as an in vitro model for small intestinal epithelium. The cells were cultured in DMEM supplemented with 10% FBS. The HaCaT human keratinocytes (CLS Cell Lines Service GmbH, Eppelheim Germany) were cultured in DMEM supplemented with 10% FBS. 184 A1N4 (a gift from M. R. Stampfer, UC Berkeley) [24] human mammary epithelial cells were cultured in a modified Improved Minimum Essential Medium (IMEM) supplemented with 0.5% FBS, 5 μg/ml recombinant human insulin (rh-insulin) (Invitrogen, Carlsbad, CA), 5 μg/ml hydrocortisone Sigma-Aldrich (St. Louis, MO), and 10 ng/ml recombinant human epidermal growth factor (rhEGF) (Promega, Madison, WI). LNCaP (ATCC, Manassas, VA) prostate cancer cells were cultured in RPMI-1640 medium supplemented with 10% FBS. The cells were incubated at 37°C in a humidified atmosphere with 5% CO_2_.

### Monoclonal antibodies

The HAI-2 monoclonal antibodies (mAbs) DC16 and XY9 were used to detect HAI-2 species with and without complex N-glycan branching, respectively [20,21]. The human prostasin mAbs YL11 and YL89 were generated using milk-derived prostasin-HAI-1 complexes as immunogen as described previously [11]. The human matriptase mAb M24 was used to detect matriptase zymogen and activated forms in complexes with protease inhibitors [25,26]. HAI-1 mAb M19, HAI-2 mAb DC16, prostasin mAb YL11, and matriptase mAb 21–9 [27] were also immobilized to Sepharose beads at 5 mg/ml gel, according the manufacturer’s instruction (GE Healthcare Life Science, Marlborough, MA).

### Western blotting

Cells were lysed in phosphate buffered saline (PBS) containing 1% Triton X-100 or in radioimmunoprecipitation assay (RIPA) buffer. Intestine tissue was lysed in RIPA buffer after being frozen in liquid nitrogen and ground with a mortar and pestle. DTNB was added to the lysis buffer to prevent cleavage of disulfide linkages [28]. Protein samples were diluted in 5x SDS sample buffer containing no reducing agent and incubated at room temperature for 5 min. Proteins were resolved by 7.5% SDS-PAGE, transferred to nitrocellulose membranes, and probed with the indicated mAbs. The binding of the mAbs was detected using HRP conjugated secondary antibodies, and visualized using the Western Lightening^®^ Chemiluminescence Reagent Plus (Perkin-Elmer, Boston, MA) and x-ray film.

### Immunopurification and immunodepletion

Immunodepletion was carried out by mixing the Caco-2 cell lysate (200 μl) with 15 μl of mAb conjugated Sepharose beads in a micro centrifuge tube that was rotated end over end in a cold room for 2 hours. The supernatant was collected after the beads were pelleted by centrifugation. For immunopurification of prostasin-HAI-1 complexes, prostasin-HAI-2 complexes, and prostasin monomers, lysate was prepared from 10 culture dishes (150 mm) of Caco-2 cells (approximately 15 ml). The lysate was then sequentially run, at a flow rate of 6 ml/hr, through three immunoaffinity columns containing respectively 1 ml M19-Sepharose (anti-HAI-1), DC16-Sepharose (anti-HAI-2), and YL11-Sepharose (anti-prostasin) beads. The beads in each column were then extensively washed with PBS containing 1% Triton X100 and the captured proteins were eluted with 0.1 M glycine buffer, with each eluted fraction being immediately neutralized by the addition of 2 M Trizma base.

### Assay for prostasin proteolytic activity

The proteolytic activity of the purified prostasin was assessed by measuring 7-Amino-4-methylcoumarin (AMC) release from a synthetic fluorogenic substrate Ac-KYHR-AMC (5 μl of 5 mM stock solution in a total volume of 200 μl) in microfluor 96-well black-wall microtiter plates (Thermo Fisher Scientific, Waltham, MA). Fluorescence from the cleaved substrate was recorded using a Wallac 1420 Victor 2 microplate reader with an excitation wavelength of 360 nm and detecting emission at 480 nm.

### Immunohistochemistry

Paraffin-embedded sections of human intestine (terminal ileum) and frozen samples of intestine tissue were obtained from the Lombardi Comprehensive Cancer Center Histopathology and Tissue Shared Resource (HTSR) at Georgetown University (http://lombardi.georgetown.edu/research/sharedresources/htsr). Tissues are collected by the HTSR from patients who have provided written consent for the use of their surplus tissues for research purposes under a Georgetown University Medical Center Institutional Review Board (IRB) approved protocol (#1992–048, PIs: Berry and Harris). Through IRB 1992–048, the HTSR has obtained informed consent from patients for the use of their excess tissue for research purposes since the implementation of HIPAA regulations in early 2003. All samples collected since then have an associated signed Informed Consent Form on file in the HTSR. The HTSR can then provide anonymous tissues to investigators without additional review. Received samples are in a de-identified form. The immunohistochemical (IHC) staining was performed as previously described [29,30]. The tissue sections were stained using the HAI-2 mAb DC16, the prostasin mAb YL89, the matriptase mAb M24, the HAI-1 mAb M19, and mouse IgG as negative control. Secondary antibody (EnVision+ Dual Link System Peroxidase) and DAB (3,3'-Diaminobenzidine) (Dako, Glostrup, Denmark) were the used for the detection of a positive signal, and cell nuclei were counterstained with hematoxylin. Images were captured using an Olympus AH2 Vanox Microscope System (Olympus, Melville, NY).

## Results

### Prostasin but not matriptase is the major HAI-2 target serine protease in Caco-2 cells

HAI-2 has been implicated in the regulation of sodium homeostasis in the GI tract, and because HAI-2 is a Kunitz-type serine protease inhibitor, it is plausible that this is mediated by the regulation of serine protease activity. Kunitz-type serine protease inhibitors suppress the enzymatic activity of their target proteases by the formation of very stable complex, as exemplified by the loss of the gelatinolytic activity of activated matriptase when in complex with HAI-1 [31]. Furthermore, these protease Kunitz inhibitor complexes are so stable that they survive SDS-PAGE when samples are run under non-reducing conditions and are not boiled prior to electrophoresis [11,32]. It is worth noting that Kunitz inhibitors cannot form stable complexes with the zymogen form of proteases due to the rapid dissociation of any interaction between the protease zymogen and protease inhibitor, yielding a very high dissociation constant (K_d_). For example, the K_d_ for binding between trypsinogen and bovine basic pancreatic trypsin inhibitor (PTI), a Kunitz-type serine protease inhibitor, at 25°C, pH 8.0 has been determined to be 2x10^-6^ M [33]. In contrast, the conformational changes associated with trypsinogen activation to trypsin dramatically increases the stability of trypsin-PTI complexes resulting in a K_d_ of 6x10^-14^ M at 25°C, pH 8.0 [34]. Thus, complexes of Kunitz inhibitors with active proteases are 10s of millions fold more stable than with protease zymogens. Thus, the detection and identification of HAI-2 complexes with a target protease demonstrates, not only the physical interaction between HAI-2 and the target protease, but also that the enzymatic activity of the protease has been inhibited by HAI-2 and the zymogen activation state of the target protease. More importantly, it provides strong evidence that a functional link between HAI-2 and its target protease is physiologically relevant when the identification and characterization of HAI-2 complex is conducted with the naturally occurring endogenous proteins both in cultured cells and human intestinal tissues.

**Fig 1:**
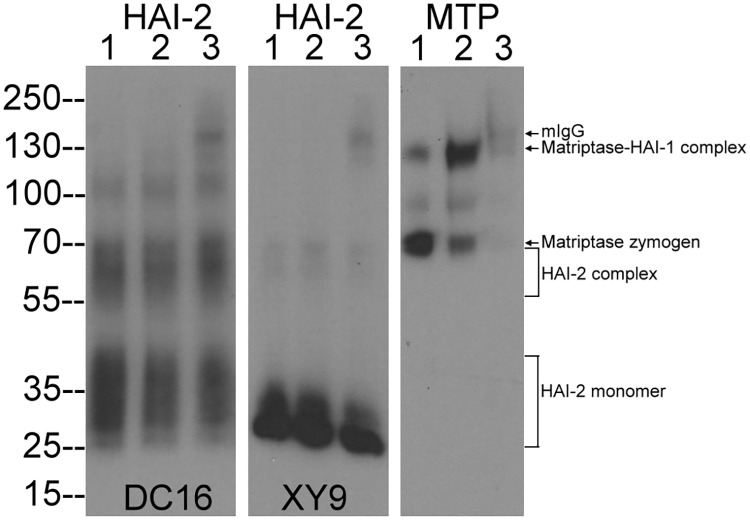
HAI-2 species in Caco-2 human enterocytes. Caco-2 human enterocytes were incubated in PBS as a non-activation control (lanes 1) or a phosphate buffer pH 6.0 for 20 min to induce matriptase zymogen activation (lanes 2). Matriptase species were subsequently immunodepleted from the lysates that had been prepared from Caco-2 cells in which matriptase zymogen activation was induced (lanes 3). The three Caco-2 lysates were then analyzed by immunoblot for HAI-2 species with significant N-glycan branching using HAI-2 mAb DC16 (HAI-2 DC16), HAI-2 species without N-glycan branching using HAI-2 mAb XY9 (HAI-2 XY9), and matriptase species using matriptase mAb M24 (Total MTP). Small quantities of mouse IgG shed from the beads used for immunodepletion can be seen as a band of approximately 150-kDa (lanes 3 in each panel) is indicated by an arrow. The HAI-2 species in Caco-2 cells have been observed in at least 10 independent experiments. The induction of matriptase zymogen activation in Caco-2 cells and the immunodepletion of matriptase species has been carried out at least 3 times. Representative data are shown.

In order to identify the HAI-2 target protease(s) potentially involved in sodium absorption, the expression of HAI-2 protein by Caco-2 cells, which have been used widely as a model for intestinal epithelia, were analyzed by immunoblot (Fig 1). Previously, we have shown that HAI-2 is expressed with two different types of N-glycan modification, which can be distinguished using two HAI-2-specific mAbs: DC16, which recognizes HAI-2 species with extensive N-glycan branching, and mAb XY9, which recognizes HAI-2 species modified with what is likely oligo mannose-type N-glycan [21]. Analysis of Caco-2 lysates with the mAb DC 16 revealed the presence of HAI-2 in three bands (Fig 1, HAI-2 DC16, lane 1). The smallest band centered round a mass of about 35-kDa is the HAI-2 monomer with extensive N-glycan branching and a diffuse appearance on immunoblot; the bands at around 65-kDa and 110-kDa may represent HAI-2 complexes with serine proteases. The mAb XY9 detected a primary band at 25-kDa, which represent the HAI-2 species without extensive N-glycan branching (Fig 1 HAI-2 XY9, lane 1). Previously we have shown that the type 2 transmembrane serine protease matriptase is a target protease of HAI-2 in breast cancer cells, the exposure of which to a pH 6.0 buffer induces extensive matriptase zymogen activation and the formation of matriptase-HAI-1 and matriptase-HAI-2 complexes [20]. When Caco-2 cells were transiently exposed to a pH 6.0 buffer, matriptase zymogen activation was induced and the 120-kDa matriptase-HAI-1 complex was formed at the cost of the matriptase zymogen which is detected as a band at 70-kDa (Fig 1, MTP, comparing lane 2 with lane 1). The induction of matriptase zymogen activation does not appear to result in the formation of detectible levels of matriptase-HAI-2 complex, nor does it alter the status of HAI-2 species detected (Fig 1 HAI-2, lanes 2). Immunodepletion of matriptase from the Caco-2 lysate removed the 70-kDa matriptase zymogen and the 120-kDa matriptase-HAI-1 complex but did not remove the HAI-2 complexes (Fig 1, lanes 3). It should be noted that the faint band at around 150-kDa in the immunodepleted samples is caused by low-level shedding of the mAb (mIgG) from the Sepharose beads which is detected by the secondary antibody (Fig 1 lanes 3). These data suggest that matriptase is not a significant HAI-2 target protease in this system, even after induction of extensive matriptase zymogen activation.

**Fig 2:**
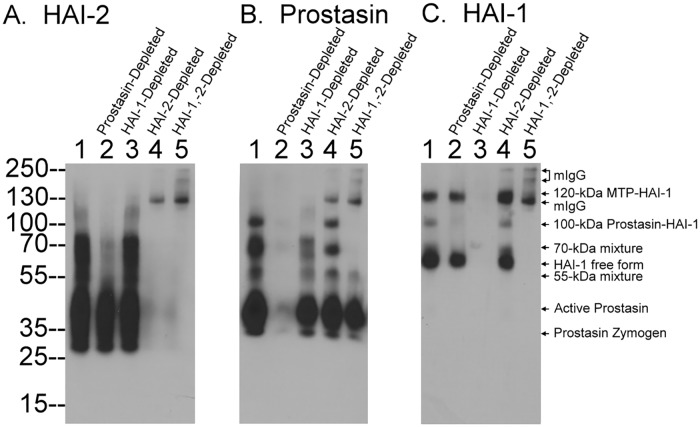
Prostasin species in Caco-2 human enterocytes. Caco-2 lysates (lanes 1) were subjected to immunodepletion to remove prostasin species (lanes 2), or HAI-1 species (lanes 3), or HAI-2 species (lanes 4), or both HAI-1 species and HAI-2 species together (lanes 5), as indicated. The lysates were analyzed by immunoblot for HAI-2 species (A.), prostasin species (B.), and HAI-1 species (C.). The identification of the prostasin species has been replicated at least twice and further verified by immunopurification in Fig 3.

We next examined whether the HAI-2 complexes contain prostasin, given that in human milk HAI-2 was found to be present in complexes with prostasin or matriptase [11]. Incubation of the cell lysate with prostasin-specific mAb YL11 linked to Sepharose resulted in the almost complete immunodepletion of the HAI-2 complexes but not HAI-2 monomer (Fig 2A, lane 2), suggesting that prostasin is in the HAI-2 complexes and represents the major target protease of HAI-2 in Caco-2 cells. The identification of prostasin as the predominant target protease of HAI-2 is consistent with the fact that HAI-2 is a very potent inhibitor of prostasin with a K*i* of 5 nM [23].

### Caco-2 cells express prostasin primarily in its activated form

When Caco-2 cell lysates were analyzed for the presence of prostasin-containing species by immunoblot with the prostasin mAb, at least five protein bands were detected, with apparent sizes of 100-, 70-, 55-, 40-, and 32-kDa (Fig 2B, lane 1). All five of these protein bands were removed when the lysate was immunodepleted using the prostasin-specific mAb-Sepharose, suggesting that these five bands are prostasin-containing species (Fig 2B, lane 2). The 100-kDa prostasin species appears to be prostasin in complex with the mature, intact 55-kDa form of HAI-1, since this complex was also detected by immunoblot with the HAI-1-specific mAb M19 (Fig 2C, lane 1). Further evidence for the identity of the 100-kDa prostasin-HAI-1 complex was additional immunodepletion-immunoblot analysis. The 100-kDa prostasin complex was immunodepleted by HAI-1-specific mAb beads (Fig 2B, comparing lane 3 with lane 1), and the 100-kDa HAI-1 complex was immunodepleted with prostasin-specific mAb beads (Fig 2C, comparing lane 2 with lane 1).

The 70-kDa prostasin species appears to be comprised of a mixture of two complexes: prostasin in a complex with a HAI-1 fragment, and prostasin in a complex with HAI-2 (Fig 2B, lane 1). Evidence for this is provided by result of immunodepletion of HAI-1 species only (Fig 2B, lane 3), or HAI-2 species only (Fig 2B, lane 4), or both HAI-1 and HAI-2 species together (Fig 2B, lane 5). The minor 55-kDa prostasin-containing species also appears to be a mixture of prostasin complexed with a HAI-1 fragment and prostasin complexed with a HAI-2 (fragment), based on the immunodepletion-immunoblot analyses (Fig 2B). These data suggest that a significant proportion of the prostasin is in complexes with HAI-1 and HAI-2 in Caco-2 cells. Both HAI-1 and HAI-2 will only bind and form stable complexes with the active form of serine proteases and not the zymogen form, and so these data also imply that a significant proportion of the prostasin is present in activated form in Caco-2 cells under regular culture conditions. It was noted that mouse IgG was again leaking from the beads during the process of immunodepletion using HAI-2 mAb. The mouse IgG was detected as three bands in the western blot analyses only in the samples with HAI-2 immunodepletion (Fig 2, lanes 4 and 5).

**Fig 3:**
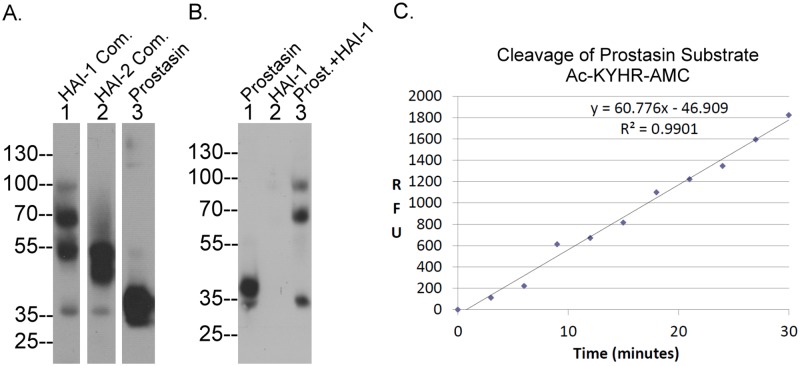
Caco-2 cells constitutively activate prostasin. A. The prostasin containing species present in Caco-2 cell lysates were separated and purified by sequential immunoaffinity chromatography using HAI-1 mAb M19-Sepharose, HAI-2 mAb DC-16 Sepharose, and then prostasin mAb YL11-Sepharose. The purified prostasin-HAI-1 complexes (lane 1), prostasin-HAI-2 complexes (lane 2), and prostasin monomers (lane 3) were analyzed for prostasin species by immunoblot using the prostasin-specific mAb YL11. B. Purified prostasin monomers (lane 1) were incubated with purified HAI-1 (lane 2) at 37°C for 5 min to allow the formation of prostasin-HAI-1 complexes (lane 3). The samples were then analyzed by western blot for prostasin-containing species. C. Purified prostasin monomers were incubated with a fluorogenic prostasin substrate and the kinetics of AMC release was recorded. Prostasin proteolytic activity assays were conducted at least four times. Representative data are shown. RFU stands for relative fluorescent unit.

In addition to its presence in the complexes, prostasin was also detected as a pair of bands with apparent masses close to the calculated molecular mass of around 35-kDa (Fig 2B, lane 1), with the minor band of 32-kDa and major band at 40-kDa, which were presumed to be prostasin “monomers”. Characterization of these species began with the separation of the monomers from the prostasin-HAI-1 and prostasin-HAI-2 complexes (Fig 3A). Prostasin-HAI-1 complexes were removed from Caco-2 cell lysate using HAI-1 mAb M19-Sepharose. The material that had bound to the column was eluted and immunoblotting for prostasin reveled three bands of 100-, 70-, and 55-kDa (Fig 3A, lane 1), consistent with the analysis of the cell lysate shown in Fig 2 (Fig 2B, lane 1), though the 100-kDa complex was somewhat degraded. Prostasin-HAI-2 complexes were next removed by passing the flow-through from the HAI-1 antibody column through a HAI-2 mAb DC16-Sepharsoe column. Analysis of the protein bound to this column revealed somewhat degraded complexes of 55-kDa (Fig 3A, lane 2), again consistent with the earlier data. A very small proportion of the previously complexed active prostasin eluted from both columns appears to have become dissociated and not re-bound to the HAI during the process of elution with pH 2.4 buffer and subsequent neutralization and can be observed as a band just above the 35-kDa marker (Fig 3A, lanes 1 and 2).

Final purification of the two prostasin monomers was achieved by immunoaffinity chromatography on a prostasin mAb YL-11-Sepharose column (Fig 3A, lane 3). To determine the activation state of the prostasin monomers, the purified prostasin monomers (Fig 3B, lane 1) were then mixed with naturally occurring, immunoaffinity purified HAI-1 (Fig 3B, lane 2) and incubated at 37°C for 10 min. This resulted in the formation of the 100-kDa and 70-kDa prostasin-HAI-1 complexes along with the disappearance of the 40-kDa prostasin monomer species, but not the 32-kDa species (Fig 3B, lane 3). As noted above, HAI-1 only binds to and forms stable complexes with active serine proteases and not with the zymogen forms of the proteases. These data, therefore, indicate that the 40-kDa prostasin monomer is active prostasin and the 32-kDa species is the zymogen form of prostasin. It is important to point out that the immunoblot assay was conducted under non-reducing and non-boiled conditions. Under these conditions the two chains of the active form of prostasin which are linked by a disulfide bond, remain attached to each other and so migrate more slowly on SDS-PAGE than the single-chain zymogen form of prostasin. The purified prostasin monomer preparation also exhibited amidolytic activity by cleaving a selective prostasin fluorogenic substrate: Ac-KYHR-AMC (Fig 3C). The proteolytic activity exhibited further supports the identification of the 40-kDa prostasin monomer as the active form of prostasin. Collectively, our data suggest that Caco-2 cells express prostasin predominantly in its activated forms, either as the free active form or in complexes with HAI-1 or HAI-2.

### Prostasin appears to be under the control of HAI-2 near the apical membrane of human intestinal enterocytes in vivo

**Fig 4:**
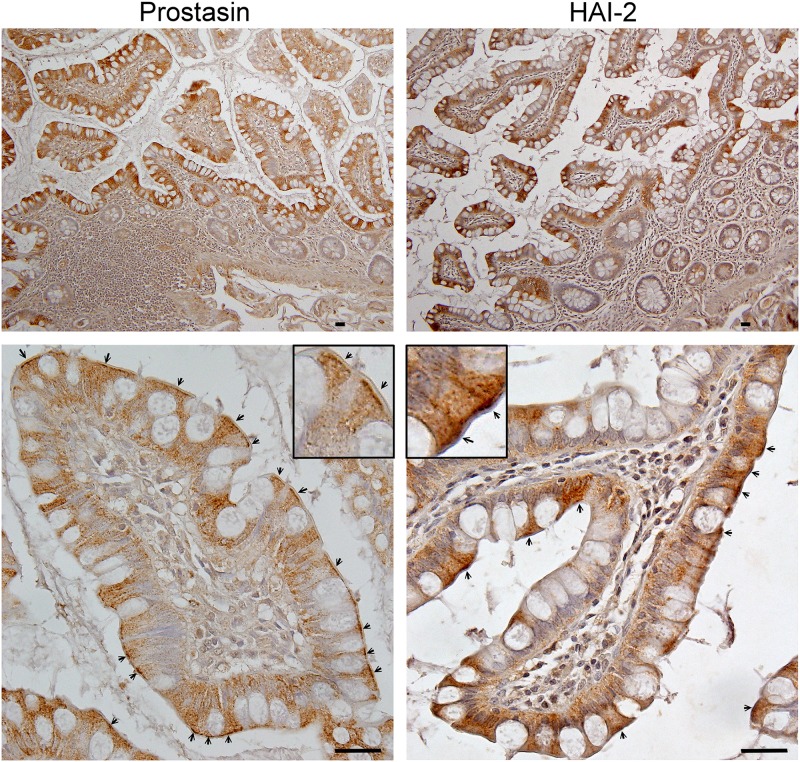
Prostasin and HAI-2 are localized intracellularly with the highest density near the apical surface of intestinal enterocytes. Tissue sections of human intestine were immunostained with the prostasin-specific mAb YL89 (Prostasin) or the HAI-2-specific mAb DC16 (HAI-2). Sections were also stained with a non-specific mouse IgG antibody as a negative control (Data not shown). Higher magnification images of the staining are presented in lower panels and the insets. Arrows indicate the accumulation of prostasin and HAI-2 right beneath brush border. Cells nuclei were counterstained blue with hematoxylin. The immunohistochemical staining studies with intestinal tissues were at least repeated twice. Representative staining is shown. Scale bar: 25 μm.

The putative functional link between HAI-2 and prostasin was further investigated by comparing the in vivo tissue distribution and subcellular localization of the proteins in the human intestine tissue by immunohistochemical (IHC) staining. Low magnification images show that prostasin immunoreactivity was detected primarily in the surface absorptive columnar epithelial cells (enterocytes) (Fig 4, Prostasin, upper panel). The signal in the crypts was much weaker, suggesting that prostasin is expressed by more differentiated enterocytes. It remains unclear whether the mucus-secreting goblet cells express prostasin due to the limited resolution of IHC (Fig 4, Prostasin, upper panel). At higher magnification (Fig 4, Prostasin lower panel), it becomes evident that prostasin is detected as punctate staining throughout the cells with much more signal focused right beneath the brush border and close to the apical plasma membrane. The distribution of HAI-2 immunoreactivity closely resembles that of prostasin, with most staining in the more differentiated enterocytes (Fig 4, HAI-2, upper panel), and as punctate staining throughout the cells with greater density toward apical surface of the cells (Fig 4, HAI-2, lower panel). The similar distribution profile of these proteins could allow HAI-2 to have access to prostasin. When paired with the immunodepletion-immunoblot analysis described above, these observations suggest that prostasin represents the major target protease of HAI-2 in the surface villus epithelial cells.

### Matriptase and HAI-1 are localized at the lateral plasma membrane of villus epithelial cells in the human intestine

**Fig 5:**
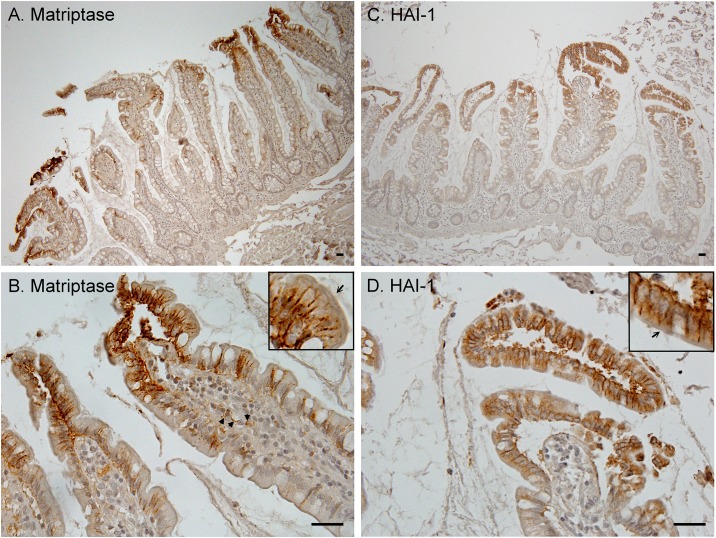
Matriptase and HAI-1 are localized at intercellular junctions between intestinal enterocytes. Tissue sections of human intestine were immunostained with the matriptase-specific mAb M24 (Matriptase) or the HAI-1-specific mAb M19 (HAI-1). Sections were also stained with a non-specific mouse IgG antibody as a negative control (Data not shown). Higher magnification images of the staining are presented in lower panels and the insets. Arrows indicate the brush border. Arrow heads indicate matriptase-positive cells in the lamina propria. Cells nuclei were counterstained blue with hematoxylin. The immunohistochemical staining studies with intestinal tissues were at least repeated twice. Representative staining is shown. Scale bar: 25 μm.

We also examined the tissue distribution and subcellular localization of matriptase and HAI-1 in human intestine by IHC, in order to provide insights into the in vivo functional relationship among the membrane-associated serine proteases and the integral membrane serine protease inhibitors. From low magnification images of this staining it appears that matriptase (Fig 5A) and HAI-1 (Fig 5C) expression is detected on the enterocytes with the greatest level of expression, particularly for matriptase, on those enterocytes that reside on the top of the villi. At higher magnification, matriptase immunoreactivity is predominantly localized at lateral plasma membrane (Fig 5B). The plasma membrane localization of matriptase was also seen for a few cells in the lamina propria (Fig 5B, arrow head). The identity of these isolated matriptase-positive cells in the lamina propria remains unclear but they may be monocytes/macrophages, some sub-populations of which express matriptase [35]. With the exception of a very few cells, matriptase was not detected beneath the brush border where we had observed that prostasin and HAI-2 were most strongly localized (Fig 4).

HAI-1 was also clearly detected at the lateral plasma membrane (Fig 5D). In addition to the sharp staining on the plasma membrane, more diffuse staining for HAI-1 was present throughout the cells, which suggests that a significant proportion of the total HAI-1 resides intracellularly. The presence of HAI-1 on the plasma membrane could allow HAI-1 to have access to matriptase and the intracellular HAI-1 could have access to prostasin. When paired with the immunodepletion-immunoblot analysis described above, these observations suggest that HAI-1 can function as an endogenous inhibitor for both matriptase and prostasin at different subcellular locations of human enterocytes.

**Fig 6:**
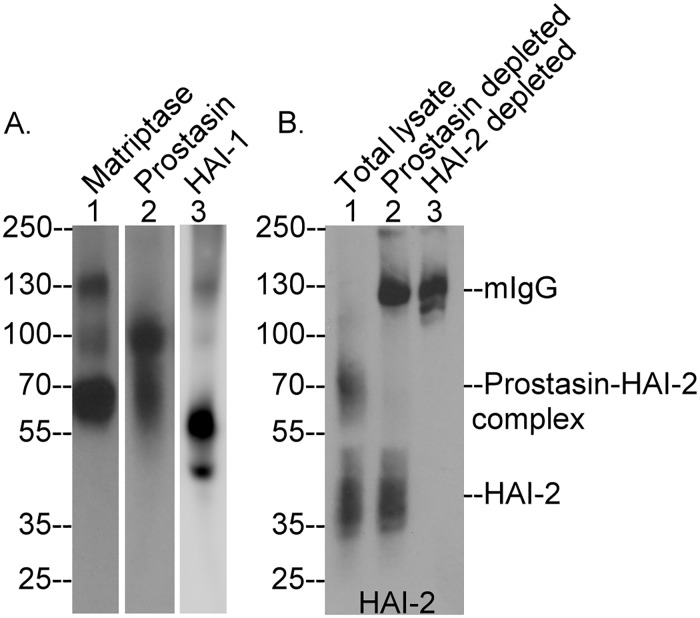
Analysis of prostasin, matriptase, HAI-1 and HAI-2 species in human intestine tissue. Human intestine tissue was used to prepare lysate that were analyzed by western blot for matriptase species (A. lane 1), prostasin species (A. lane 2), HAI-1 species (A. lane 3) and HAI-2 species (B. lane 1). Various amounts of tissue proteins were analyzed for the four proteins examined. The lysate was then immunodepleted by incubation with prostasin-specific mAb YL-11-Sepharose or HAI-2-specific mAb DC16-Sepharose. The immunodepleted lysate was analyzed by western blot for HAI-2 species (B. lanes 2 and 3). Mouse IgG (mIgG) that leaked from the beads, prostasin-HAI-2 complex, and HAI-2 were indicated. Human intestine tissues were examined at least twice with the same expression status observed for the four proteins.

The biochemical nature of the matriptase, prostasin, HAI-1 and HAI-2 present in human intestine tissue was next analyzed by immunoblot assays conducted on lysates prepared from frozen human tissues (Fig 6). Matriptase was detected primarily as the 70-kDa zymogen form and to a lesser degree in a 120-kDa complex with HAI-1 (Fig 6A, lane 1), a situation that is similar to that observed in Caco-2 cells (Fig 1). A minor 100-kDa matriptase-containing species was also detected (Fig 6A, lane 1). Its identity remains unclear, whereas it could be a degraded product of the 120-kDa matriptase-HAI-1 complex. Prostasin was detected as the 100-kDa complex with HAI-1 and the 70-kDa complex with HAI-2 (Fig 6A, lane 2), both of which were also detected in Caco-2 cells (Figs 1 and 2). While free active prostasin was not detected in the intestine tissue lysate, the observation that essentially all of the prostasin detected was in the activated form in the intestine tissue lysate, strongly resembles the situation in Caco-2 cells in which very little prostasin zymogen was detected. The high activation status of the prostasin is very different from the situation with matriptase: the majority of which was in the zymogen form. The presence of 120-kDa matriptase-HAI-1 complex and 100-kDa prostasin-HAI-1 complex in the tissue lysate was also confirmed by the detection of both complexes using the HAI-1 mAb (Fig 6A, lane 3). The majority of HAI-1 was detected in its free form (Fig 6A, lane 3), consistent with its high-level abundance relative to the target proteases as we have previously shown [36]. HAI-2 was detected in both the free and complexed forms in roughly equal amounts (Fig 6B, lane 1). The HAI-2 complex can be immunodepleted from the tissue lysate by both the prostasin mAb (Fig 6B, lane 2) and the HAI-2 mAb (Fig 6B, lane 3), confirming the presence of the prostasin-HAI-2 complex in human intestine tissue. Collectively, these data show that the expression and zymogen activation status of the two membrane-associated serine proteases and their relationship with the two Kunitz-type serine protease inhibitors is very similar in the human intestine tissue that observed in the Caco-2 cells.

### HAI-1 is a universal prostasin inhibitor whereas prostasin inhibition by HAI-2 appears to be cell-type selective

**Fig 7:**
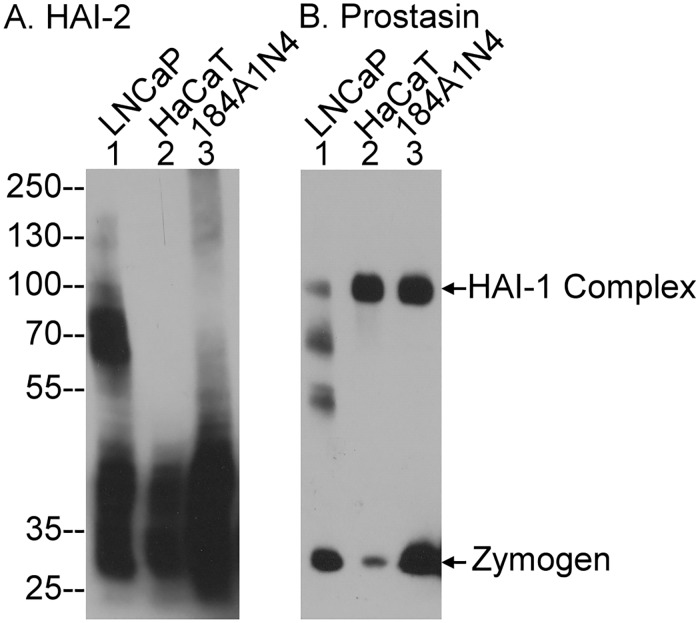
Analysis of the prostasin and HAI-2 species present in human prostate cancer cells, keratinocytes and mammary epithelial cells. LNCaP human prostate cancer cells, HaCaT human keratinocytes and 184 A1N4 human mammary epithelial cells were transiently exposed to a pH 6.o buffer to induce matriptase and prostasin zymogen activation. Lysates were prepared and analyzed by western blot for HAI-2 species using the HAI-2 specific mAb DC16 and for prostasin species using the prostasin-specific mAb YL11. Western blot analysis for the prostasin and HAI-2 species in these three cell lines has been conducted multiple times (>5) with the same profile observed. Representative data are shown.

In enterocytes, prostasin activity is under the control of both HAI-1 and HAI-2. These three proteins are, however, widely expressed by a variety of epithelial tissues, and so we next set out to examine the functional relationship between prostasin and the HAIs in different types of epithelial cells, including LNCaP prostate cancer cells, HaCaT immortalized human keratinocytes, and 184 A1N4 mammary epithelial cells. Cells from all three lines were transiently exposed to a pH 6.0 buffer to induce zymogen activation of prostasin and matriptase [37]. HAI-2- and prostasin-containing species were then examined by western blot with antibodies against these proteins (Fig 7). For the HAI-2 species, LNCaP cells, but not the HaCaT or the 184 A1N4 cells, resemble Caco-2 cells with respect to the presence of significant levels of a 70-kDa HAI-2 complex, in addition to the free form of HAI-2 (Fig 7A). For the prostasin species, in addition to the zymogen form, three prostasin species with sizes of 100-, 70- and 55-kDa were detected (Fig 7B, lane 1). In contrast, the 100-kDa prostain-HAI-1 complex was detected as the predominant type of prostasin complex in the human keratinocytes and in the human mammary epithelial cells (Fig 7B, lanes 2 and 3). These data suggest that while inhibition of prostasin by HAI-1 may be a relatively standard event in the cells that express both proteins, the role of HAI-2 in prostasin inhibition may be rather more cell-line, or cell-type selective.

## Discussion

The aberrant regulation of HAI-2 target protease(s) may be the underlying mechanism responsible for the sodium loss that seems to be at the heart of the primary symptom of patients with SCSD [3]. The detection of activated prostasin in complex with HAI-2 in lysates prepared from human intestine tissue and from Caco-2 cells identifies prostasin as the predominant physiologically relevant target protease of HAI-2 in human enterocytes. In addition, vast majority of prostasin present in activated form in complex with HAI-1 or HAI-2 in intestinal tissue indicates prostasin proteolysis is constitutively active and under tight control. The loss of HAI-2 function in patients with SCSD would, therefore, be expected to result in aberrant regulation of prostasin, which may subsequently contribute to the sodium loss seen in these patients, although the underlying mechanism remains to be further investigated. Furthermore, the high zymogen activation state of prostasin and the selective importance of the inhibition of prostasin by HAI-2 observed in enterocytes may explain why this cell type is more susceptible than other epithelial tissues to loss of function mutations of HAI-2. Given that HAI-2 contains two Kunitz domains. Kunitz domain 1 is responsible for the binding and inhibition of active prostasin. Kunitz domain 2 could also have its own target protease. Indeed, a 110-kDa HAI-2 complex was also detected in Caco-2 cells (Figs 1 and 2), which is greater than the 65-kDa prostasin-HAI-2 complex and may contain an as yet unidentified protease. Whether and how this and other putative HAI-2 target proteases are dysregulated by HAI-2 mutations and subsequently contribute to pathogenesis of SCSD can be determined only after they are identified. Although we have established the relationship between HAI-2 and prostasin, the possibility remains that the impact of HAI-2 mutation on SCSD is mediated by altered activity against a different client protease.

Excessive proteolysis of HAI-2 target protease(s) has been proposed to lead to the failure of sodium absorption in SCSD patients [3]. There is no evidence of primary structural abnormalities in the intestinal epithelium of SCSD patients. The identification of prostasin as the major target protease of HAI-2 in human enterocytes suggests that the putative excessive prostasin proteolytic activity could, therefore, alter intestinal epithelial absorption or secretion of sodium by altering the activity of a variety of transport mechanisms, including co-transporter, coupled exchangers, and the epithelial sodium channel (ENaC). Among these transport mechanisms, the activity of ENaC is tightly regulated by proteolysis. Many membrane-associated serine proteases, including prostasin and matriptase, have been shown to increase ENaC activity when these proteases and ENaC are exogenously expressed in *Xenopus laevis* oocytes [38–40]. Furthermore, this protease-induced ENaC activity can be suppressed by HAI-2 [40]. While this functional analyses support the role of proteolysis in sodium absorption, ENaC is likely not the primary transport mechanism which is affected by HAI-2 mutations and is responsible for the failure of sodium absorption seen in SCSD patients. Firstly, loss of or decreased HAI-2 inhibitory activity caused by the mutations identified in SCSD patients is likely to increase prostasin proteolytic activity, which conceivably could subsequently increase ENaC activity and sodium absorption rather than causes the failure of sodium absorption. Secondly, while proteolytic cleavage is involved in ENaC activation, prostasin proteolytic activity is dispensable for the induction of enhanced ENaC activity in the oocytes model. Addition of catalytic triad mutant prostasin also induces enhanced ENaC activity [39]. It has been suggested that an as yet to be identified endogenous oocyte protease interacts with prostasin zymogen to mediate the activation of ENaC [41]. Thirdly, the reported functions of the zymogen form of prostasin, including ENaC activation, may not be physiologically relevant with respect to the mechanism underlying the failure of sodium absorption in SCSD patients since it appears that the vast majority of prostasin present in human intestine tissue is activated. Thus, it is very difficult to reconcile the high levels of constitutive zymogen activation present in vivo and the putative functions of prostasin zymogen identified via genetic manipulation in various model systems [39,42–45].

In a study by Szabo et al., [46], the formation of a stable prostasin zymogen complex with HAI-2 was also reported. In that study recombinant human prostasin was prepared using 293T cells and was considered to all be in the zymogen form based on: 1) the lack of binding of the protein to PN-1 and 2) the larger size of the material than active prostasin [47]. When mixed with HAI-2, a complex was formed, leading Szabo et al to conclude that prostasin zymogen, like active prostasin, can form a stable complex with HAI-2. While both criteria for concluding that the recombinant prostasin was in the zymogen form seem convincing at first glance, we have found them to be problematic. Prostasin zymogen is a single-chain molecule whereas active prostasin contains a light chain with 12 amino acid residues and a heavy chain, held together by a disulfide bond. Removal of the light chain by breaking the disulfide bond will liberate the prostasin heavy chain, which is smaller than prostasin zymogen by 12 amino acid residues. The limited resolution of SDS-PAGE and the presence of at least two prostasin species, which are likely the result of posttranslational modifications, make it very difficult to distinguish active prostasin from zymogen prostasin by immunoblot analysis [43]. Nevertheless, the specification of zymogen versus active prostasin by size difference has been also used in several previous studies [46–48]. In the study by Szabo et al., [46], the samples for immunoblot analysis were treated with reducing agent in order to dissociate active prostasin into the heavy chain and light chain. The samples were, however, not heat-treated as the immunoblot analysis was intended to detect prostasin-HAI-2 complexes. Thus, the authors were trying to achieve the delicate balancing act of reducing the disulfide bond linking the heavy and light chains, without reducing the disulfide bonds on which the structure of the prostasin and ability to form a complex with HAI-2 depends. This presents the significant challenge of selecting the correct temperature, concentration of reducing agent, and incubation time to selectively break one disulfide bond and not the others. Given that prostasin-HAI-2 complexes were clearly detected in the Szabo et al., study, the conditions chosen were sufficiently mild to avoid breaking those disulfide bonds required for complex formation and so it seems inadvisable to conclude that dissociation of the heavy and light chains has occurred without direct evidence for the presence of the free light chain which is not presented. This weakens the conclusion that the recombinant prostasin present in one complex must be in the zymogen form, and that the matriptase-treated prostasin in the other complex must be activated, based on the size difference observed, particularly considering the tricky, non-standard experimental conditions employed.

The inability of the recombinant prostasin to form a complex with PN-1 seems, on its face, to be good evidence for the conclusion that the recombinant prostasin is in the zymogen state in the Szabo et al. study [46]. This observation could, however, be the result of the low complex forming efficiency of recombinant PN-1. For example, when the recombinant PN-1 was incubated with matriptase-activated active prostasin, less than half of the active prostasin was detected in a complex with PN-1. In contrast, all of the active prostasin was complexed with HAI-2 when the matriptase-activated active prostasin was incubated with HAI-2. Previously, the recombinant prostasin expressed in and prepared from 293-EBNA cells was showed to contain active prostasin, which can form stable complexes with PN-1 [49,50]. It seems highly likely, therefore, that the recombinant prostasin from 293T cells used by Szabo et al., [46] is also a mixture containing both prostasin zymogen and active prostasin. Thus, the complex formed when the recombinant prostasin was incubated with HAI-2 in the study resulted from the interaction of HAI-2 with enzymatically active prostasin and not the zymogen form. This explanation of the result in Szabo et al., [46] is consistent with the long-held view that only active serine proteases and not the zymogen forms of those proteases can form stable complex with Kunitz-type inhibitors [33,34].

The problematic conclusion that the zymogen form of prostasin can form stable complexes with protease inhibitors is not limited to HAI-2. In a study by Crisante et al. [42] recombinant prostasin with mutations at all three amino acids of the active site triad was reported to form a stable complex with PN-1, a serpin-type inhibitor. Serpin inhibitors inactivate active proteases by the formation of acyl-enzyme intermediate involving the serine residue in the active site triad [51], and so PN-1 should not be able to form a stable complex with the mutant prostasin. This suggests that the 75-kDa SDS-stable complex observed by Crisante et al., is not a PN-1-mutant prostasin complex. Indeed, in the Szabo et al., study [46], mutation at the serine residue in the active site triad of prostasin abolished the formation of a stable complex with PN-1 [46]. This adds weight to the argument that wild-type recombinant prostasin used by Szabo et al was not in the zymogen form, but rather was at least partially activated as has been seen by others when the protein is expressed in 293 cell-based systems.

The IHC staining data clearly indicate that prostasin is primarily present in intracellular granules/vesicles. Immunoblot analysis further indicates that vast majority of prostasin present in the cells has been activated and is present in complexes with HAI-2 or HAI-1 in human enterocytes in vivo. The intracellular granules/vesicles which are likely within the secretory pathway, therefore, represent the regulatory and functional location where prostasin executes its biological function. Thus, zymogen activation, the action of the protease on downstream substrates, and the HAI-mediated inhibition of prostasin likely take place intracellularly. The putative excessive prostasin proteolytic activity in the secretory pathway resulting from the loss of functional HAI-2 may, therefore, simply enhance the processing and/or activation of the normal prostasin downstream substrates. Alternatively, the excessive prostasin proteolytic activity might result in the non-specific cleavage of proteins in the secretory pathway that are not usually prostasin substrates when HAI-2 is present to moderate prostasin activity. This might negatively impact sodium absorption and secretion by impairing the function of the secretory pathway and indirectly affecting the trafficking of a variety of sodium transporters, important mechanisms for regulation of the activities of ion transporters. The indirect mechanism is supported by the overlapping and compensatory nature of the many ion transporters involved in sodium absorption in the intestine [52]. For example, NHE-3 is the dominant Na^+^/H^+^ exchanger, but targeted deletion of NHE-3 causes only slight diarrhea due to compensation for the loss of NHE3 function by upregulated expression of ENaC in the colon of NHE-3 knockout mice [53]. It appears, therefore, less likely that dysregulation of a single ion transporter by the increased activity of a HAI-2 target protease could cause the severe failure in sodium absorption. The hypothesis that it is excessive proteolysis caused by the loss of protease inhibitor that is responsible for the defect in these patients is supported by the observation that while matriptase, HAI-1, and HAI-2 play dispensable roles in placenta development, the defect in placental development caused by targeted deletion of HAI-1 or HAI-2 can be rescued by simultaneous deletion of matriptase [13,15].

In solution-based experiments, both HAI-1 and HAI-2 are potent inhibitors of matriptase and prostasin [11,20]. At the cellular level, however, the roles of HAIs in the control of matriptase and prostasin are much more complicated and largely depend on whether the proteins are coincided [20]. The inhibition of matriptase by HAI-1 appears to be a ubiquitous process across different types of epithelial and carcinoma cells. The data generated here, with an admittedly limited number of cell lines, suggests that HAI-1 may also be a common inhibitor for prostasin. The IHC staining data indicate that HAI-1 regulates matriptase function at cell-cell junctions and prostasin function inside the cells. Prostasin inhibition by HAI-2 is also an intracellular event in human enterocytes, based on their localization near the apical surface right beneath the brush border and the activated status of the majority of the prostasin present in the intestine tissues. In contrast to human enterocytes, the inhibition of prostasin by HAI-2 appears to be negligible in human keratinocytes and mammary epithelial cells. The inhibition of prostasin by HAI-2, therefore, appears to be a more selective rather than a ubiquitous process. The selective inhibition of prostasin by HAI-2 may explain the selective impact of HAI-2 mutation which appears to be limited to few organs, primarily the GI tract and cornea in spite of the broad expression of both HAI-2 and prostasin in a variety of epithelial tissues.

In summary, prostasin but not matriptase has been shown to be the target protease of HAI-2 in human enterocytes *in vivo* in the GI tract and *in vitro* in Caco-2 cells. The subcellular localization of prostasin near the apical surface and the zymogen activation and protease inhibition status of the enzyme indicates that prostasin probably functions as an intracellular protease under the tight control of HAI-1 and HAI-2, and that it most likely acts on substrates inside the cells. Prostasin undergoes constitutive, robust zymogen activation through a process that does not involve matriptase, which is primarily localized at the lateral plasma membrane. Our study provides insights to the molecular and cellular mechanism underlying the regulation of membrane-associated proteolysis in enterocytes, dysregulation of which may affect sodium homeostasis as seen in SCSD patients.

## Abbreviations

AMC: 7-Amino-4-methylcoumarin
CSD: congenital sodium diarrhea
GI: gastrointestinal
HAI: hepatocyte growth factor activator inhibitor
SCSD: syndromic congenital sodium diarrhea
